# Engineered Fibroblast Extracellular Vesicles Attenuate Pulmonary Inflammation and Fibrosis in Bleomycin-Induced Lung Injury

**DOI:** 10.3389/fcell.2021.733158

**Published:** 2021-09-23

**Authors:** Abdulrahman Ibrahim, Alessandra Ciullo, Chang Li, Akbarshakh Akhmerov, Kiel Peck, K. C. Jones-Ungerleider, Ashley Morris, Alberto Marchevsky, Eduardo Marbàn, Ahmed Gamal Ibrahim

**Affiliations:** ^1^Faculty of Medicine, University of Queensland/Ochsner Clinical School, New Orleans, LA, United States; ^2^Smidt Heart Institute, Cedars Sinai Medical Center, Los Angeles, CA, United States; ^3^Pulmonary Pathology, Cedars Sinai Medical Center, Los Angeles, CA, United States

**Keywords:** extracellular vesicles (EVs), genetic engineering, regenerative therapy, lung injury, inflammation, fibrosis

## Abstract

Pulmonary fibrosis is a progressive disease for which no curative treatment exists. We have previously engineered dermal fibroblasts to produce extracellular vesicles with tissue reparative properties dubbed activated specialized tissue effector extracellular vesicles (ASTEX). Here, we investigate the therapeutic utility of ASTEX *in vitro* and in a mouse model of bleomycin-induced lung injury. RNA sequencing demonstrates that ASTEX are enriched in micro-RNAs (miRs) cargo compared with EVs from untransduced dermal fibroblast EVs (DF-EVs). Treating primary macrophages with ASTEX reduced interleukin (IL)6 expression and increased IL10 expression compared with DF-EV-exposed macrophages. Furthermore, exposure of human lung fibroblasts or vascular endothelial cells to ASTEX reduced expression of smooth muscle actin, a hallmark of myofibroblast differentiation (respectively). *In vivo*, intratracheal administration of ASTEX in naïve healthy mice demonstrated a favorable safety profile with no changes in body weight, lung weight to body weight, fibrotic burden, or histological score 3 weeks postexposure. In an acute phase (short-term) bleomycin model of lung injury, ASTEX reduced lung weight to body weight, IL6 expression, and circulating monocytes. In a long-term setting, ASTEX improved survival and reduced fibrotic content in lung tissue. These results suggest potential immunomodulatory and antifibrotic properties of ASTEX in lung injury.

## Introduction

Pulmonary fibrosis (PF) is a progressive lung disease characterized by alveolar epithelial damage and the accumulation of collagen in the pulmonary interstitium (Barratt et al., 2018). Globally, PF is on the rise with incidence and prevalence nearly doubling (Hutchinson et al., 2015; Strongman et al., 2018) with commensurate increases in resource use and cost (Diamantopoulos et al., 2018). Current strategies include antifibrotic compounds and tyrosine kinase inhibitors such as pirfenidone and nintedanib, respectively (Raghu et al., 2015). Despite these advances, options remain limited as current treatment strategies only function to decelerate disease progression.

Extracellular vesicles (EVs) are nanosized lipid bilayer particles secreted by nearly all cell types and function as a versatile mode of paracrine and endocrine signaling (Sahoo et al., 2021). EVs derived from therapeutic cell types are the principal signaling mediators of cell therapy in a variety of models (Marban, 2018b; Sahoo et al., 2021). Therapeutic EVs have the potential to repair tissue in ways conventional therapeutics cannot. This is due, in part, to the plethora of bioactive signals in their payload which affect multiple pathways (as opposed to a single target) (Marban, 2018a; Popowski et al., 2020). Our group has recently identified pathways involved in cell (and EV) potency in a therapeutic cell type, cardiosphere-derived cells (CDCs), a cardiac progenitor cell type with preclinically and clinically demonstrated tissue-reparative capacity (Smith et al., 2007; Makkar et al., 2012; Gallet et al., 2016). This finding led to engineering these therapeutic properties in an otherwise non-therapeutic cell type, skin fibroblasts. Constitutive activation of beta-catenin and gata4 in skin fibroblast turned them into producers of therapeutic EVs.

We call these engineered fibroblasts and the EVs they produce activated specialized tissue effector cells (ASTECs) or extracellular vesicles (ASTEX). Preclinical data suggest that ASTECs and ASTEX recapitulate the therapeutic effects of CDCs and CDC-EVs. Previous work demonstrates that ASTECs and ASTEX trigger functional improvement in injured mouse hearts (Ibrahim et al., 2019). Furthermore, in a mouse model of Duchenne muscular dystrophy (DMD), ASTEX trigger exercise improvement, attenuation of fibrosis, and preservation of skeletal muscle myofibers (Ibrahim et al., 2019). Further investigation implicated enrichment of miR-92a in ASTEX and downstream signaling of the bone morphogenic protein among the protissue reparative pathways. Bone morphogenic protein (BMP) signaling plays an active role in lung tissue recovery. For instance, upregulation of BMP signaling through derepression of noggin was protective in a mouse model of bleomycin-induced lung injury (De Langhe et al., 2015). Furthermore, single-cell analysis of the injured lung epithelium identified BMP repression and its rescue-attenuated epithelial metaplasia and fibrosis (Cassandras et al., 2020). Finally, epigenetic analysis of lung tissue from pulmonary fibrosis patients identified significant methylation of the miR-17-92 cluster (of which miR-92a is included) (Dakhlallah et al., 2013). Among the targets of miR-92a is WNT1-inducible-signaling pathway protein 1 (WISP1) that signals to the profibrotic transforming growth factor-beta (TGFβ) pathway in pulmonary fibrosis (Berschneider et al., 2014). Given this mechanistic rationale, we investigated the therapeutic utility of ASTEX *in vitro* and in a bleomycin mouse model of lung injury.

## Results

### Engineered Fibroblasts Secrete Higher Quantities of EVs With Wider Size Distribution and Distinct Marker Expression From Primary Fibroblast EVs

**FIGURE 1:**
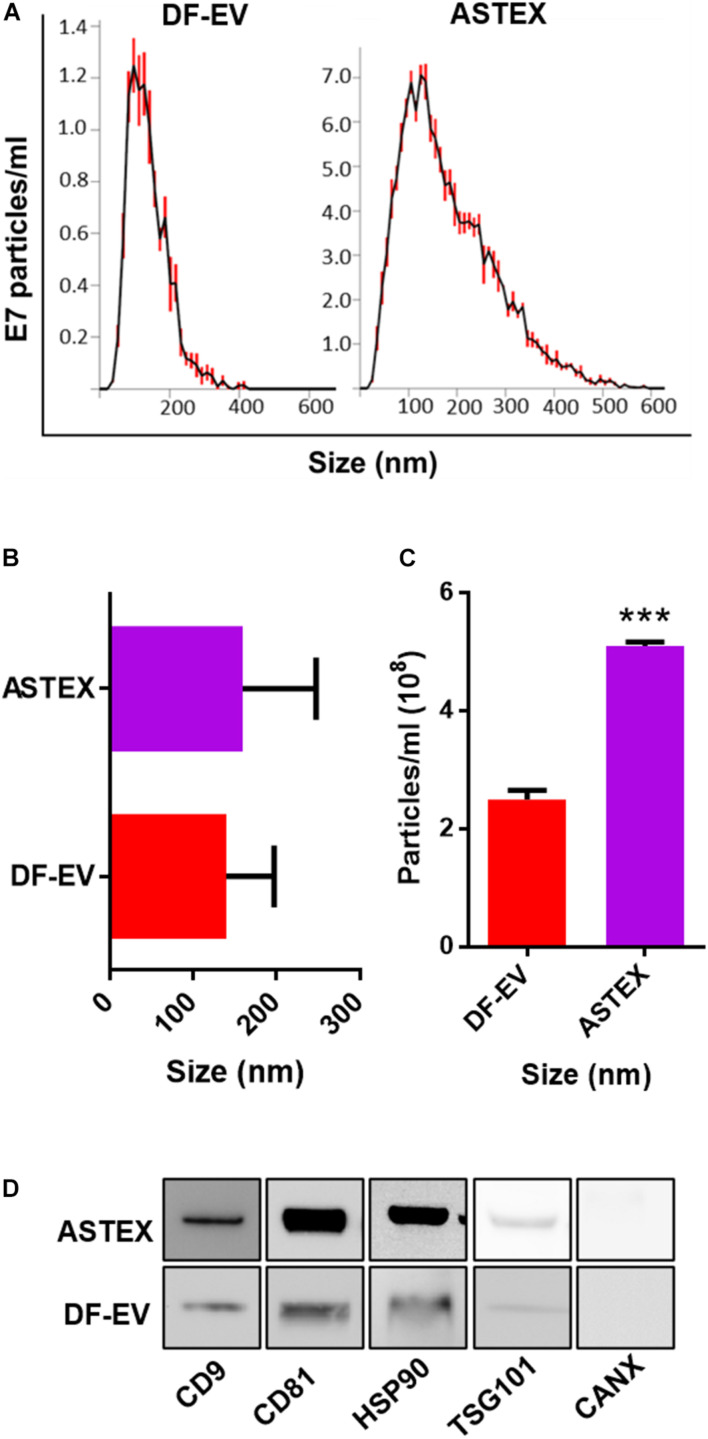
Characterization of DF-EVs and ASTEX. **(A–C)** Nanosight tracking analysis showing size distribution and concentration from conditioned media. **(D)** Western blot of conserved EV markers including tetraspanins (CD9 and CD81), heatshock protein 90 (HSP90), tumor susceptibility gene 101 (TSG101), and absence of the ER protein calnexin (CANX). Statistical comparison was done using independent Student’s *t*-test with 95% CI; *p < 0.05, **p < 0.01, ***p < 0.001.

Following transduction of dermal fibroblasts with beta-catenin and gata4, ASTECs exhibited features of cell immortalization including faster growth rate and extended population doubling and increased telomerase (Ibrahim et al., 2019). Nanosight tracking analysis demonstrated that ASTEX had a broader diversity of EV sizes compared with EVs from primary dermal fibroblasts (DF-EVs) as shown by nanosight tracking analysis (DF-EV: Figure 1A). Despite this, the average size difference between DF-EVs and ASTEX was nominal (Figure 1B). By quantity, ASTECs secrete nearly double the amount of EVs compared with primary fibroblasts (Figure 1C). This was expected as immortalization has been shown to increase EV secretion (Yu et al., 2006). Analysis of EV preparations confirmed the presence of conserved EV markers including CD9, CD81, HSP90, and TSG101 and the absence of the contaminating endoplasmic reticulum marker calnexin (Figure 1D).

### The ASTEX Proteome Is Enriched in Transcription Factors and Deficient in FGF Signaling and Complement Activators Compared to Fibroblast EVs

**FIGURE 2:**
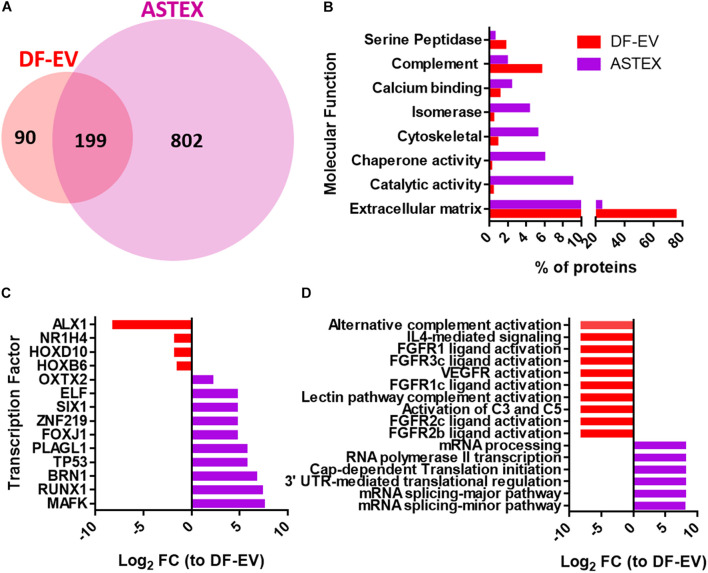
Dermal fibroblast EV (DF-EV) and ASTEX proteomic analysis. **(A)** Venn diagram showing abundance of proteins in unique and common proteins found in DF-EVs and ASTEX. **(B)** FunRich analysis of proteins with molecular function **(C)**, transcription factor, and biological pathway **(D)**.

To compare the protein content of DF-EVs and ASTEX, we performed proteomics on both EV populations. Using FunRich (Pathan et al., 2015), a software tool for functional enrichment and network analysis, we identified clear differences in protein content. ASTEX contained a wider diversity of proteins (Figure 2A). ASTEX had lower levels of extracellular matrix and complement factors and were enriched in proteins with enzymatic activity and chaperone proteins (Figure 2B). Among the most bioactive protein cargo in EVs are transcription factors as they can affect multiple gene targets in host cells. Analysis of transcription factors in both groups showed that ASTEX contained a wider diversity of transcription factors activated by beta-catenin and gata4 including MAFK (Katsuoka et al., 2000; Cao et al., 2019) and RUNX1 (Su and Gudas, 2008; Ugarte et al., 2015) which are involved in antioxidant response (Nguyen et al., 2000) and regulation of hematopoiesis (Bowers et al., 2010; Figure 2C). ASTEX were also deficient in homeobox proteins most notably ALX1, which are downregulated by beta-catenin (Ettensohn et al., 2003). Analysis of the predicted biological activity of EV proteins demonstrated suppression of pathways downregulated by beta-catenin signaling including vascular endothelial growth factor (VEGFR) (Zhang et al., 2001) and fibroblast growth factor signaling (FGFR) (Wang et al., 2017) in ASTEX compared with DF-EVs. The most prominent biological activity in ASTEX was relevant to mRNA transcription and translation (Figure 2D). Beta-catenin activation is associated with broad changes in the transcriptome with a corresponding increase in protein translation (Valenta et al., 2012). Therefore, ASTEX had reduced complement fixation capacity (and immune activation), less extracellular matrix burden, and increased catalytic activity and mRNA transcription. These results suggest fundamental proteomic changes in the beta-catenin/gata4-engineered fibroblasts and, by extension, their EVs.

### ASTEX Are Enriched in miRNAs With Anti-Inflammatory and Anti-Fibrotic Function

**FIGURE 3:**
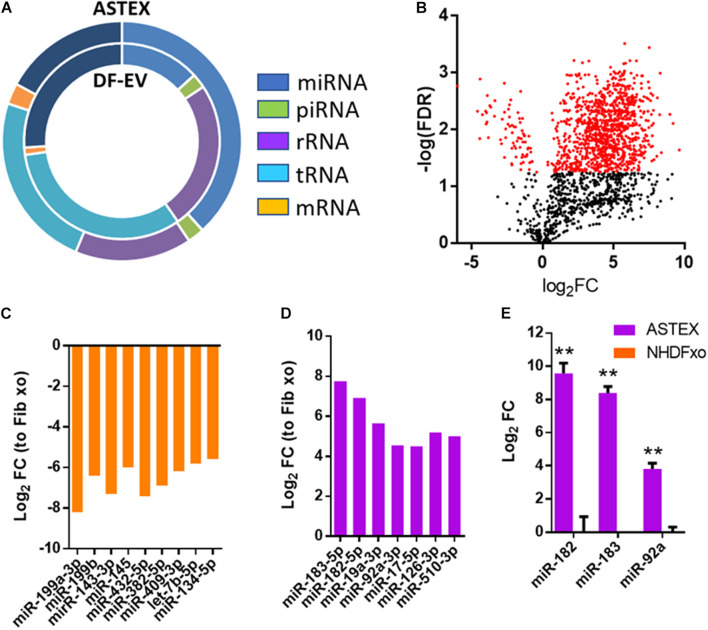
Dermal fibroblast EV (DF-EV) and ASTEX proteomic analysis. **(A)** Distribution of RNA classes in EV-RNA of DF-EV and ASTEX. **(B)** Volcano plot showing differential expression of miRs expressed as fold change of ASTEX over DF-EV. **(C)** MiRs enriched in DF-EVs compared with ASTEX and **(D)** miRs enriched in ASTEX compared with DF-EVs. **(E)** QPCR validation of miRs enriched in ASTEX. Statistical comparison was done using independent Student’s *t*-test with 95% CI; *p < 0.05, **p < 0.01, ***p < 0.001.

Sequencing RNA from DF-EVs and ASTEX showed dramatic changes in RNA content. Most strikingly, activation of beta-catenin and gata4 in fibroblasts increased micro-RNA (miR) and Piwi RNA (piRNA) abundance in ASTEX. MiR abundance, in particular, increased nearly threefold compared with DF-EVs. Moreover, the abundance of ribosomal RNA (rRNA) and transfer RNA (tRNA) were reduced in ASTEX (Figure 3A). An analysis of differentially expressed miRs identified significant divergence in the miR content of DF-EVs and ASTEX (Figure 3B). ASTEX were deficient in proinflammatory and profibrotic miRs including miR-199a, miR-143, miR-382, and miR-134 (Figure 3C). MiR-199a promotes fibrosis through suppression of caveolin and induction of the TGFβ pathway (Yang et al., 2020). MiR-143 also promotes fibrosis through targeting sprouty RTK signaling antagonist 3 (Spry3) to activate the p38-ERK-JNK signaling axis (Li et al., 2019). MiR-382 promotes inflammation and fibrosis through targeting of PTEN and potentiation of the NFkB-AKT axis (Wang et al., 2020). MiR-134 promotes inflammatory activation in macrophages through targeting angiopoietin-like 4 (Lan et al., 2016). Conversely, ASTEX were enriched in immunoregulatory and antifibrotic miRs including miR-183, miR-182, miR-19a, and miR-92a (Figure 3D). MiR-183 and miR-182 target epithelial to mesenchymal transition and myofibroblast development through targeting snail family transcriptional repressor 1 (SNAIL) (Li et al., 2018) and C/EBP homologous protein (CHOP), respectively (Yao et al., 2017; Burman et al., 2018). MiR-19a regulates inflammation through targeting of suppressor of cytokine signaling 1 (SOCS1), a potentiator of macrophage activation (Gao et al., 2019). Finally, miR-92a, a miR identified (and discussed earlier) as a driver of beta-catenin-mediated therapeutic potency (Ibrahim et al., 2019), regulates macrophage inflammation through attenuation of mitogen-activated protein kinase kinase 4 (MKK4) (Lai et al., 2013) and fibrosis through targeting of the TGFβ mediator, WISP1 (Berschneider et al., 2014). Finally, we validated the overexpression of these therapeutic miRs by qPCR to demonstrate significant enrichment in ASTEX compared with DF-EVs (Figure 3E). Therefore, ASTEX contain miRs that target inflammatory and profibrotic pathways compared with DF-EVs.

### ASTEX Exert Immunomodulatory and Anti-Fibrotic Effects *in vitro*

**FIGURE 4:**
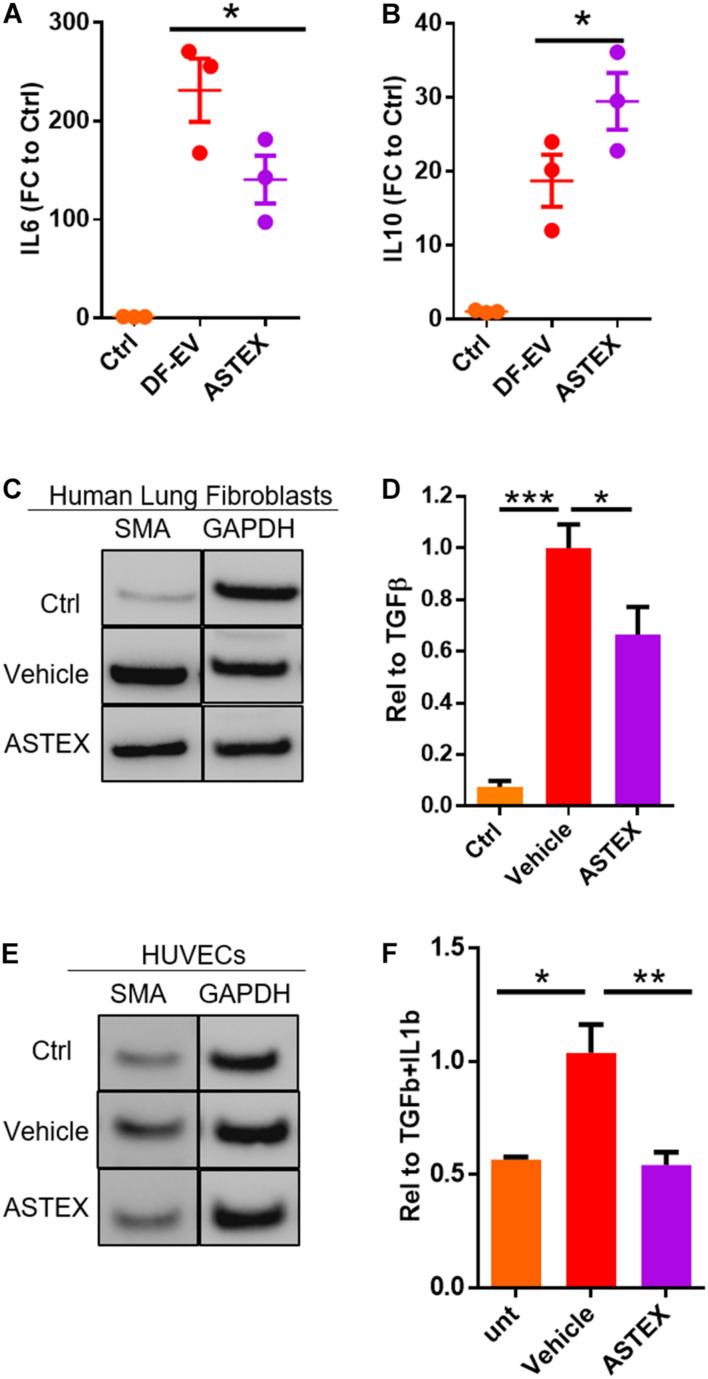
ASTEX are immunomodulatory and antifibrotic *in vitro*. Macrophages incubated with ASTEX have reduced levels of IL6 expression **(A)** and increased IL10 **(B)** (each point represents one rat donor in technical duplicates) compared with DF-EVs. **(C,D)** Attenuation of fibroblast activation in human lung fibroblasts by ASTEX as shown by reduced expression of smooth muscle actin (*n* = 3 biological replicates). **(E,F)** Attenuation of endothelial to mesenchymal transition in human umbilical vein endothelial cells (HUVECS; *n* = 3 biological replicates). Statistical analysis done using one-way ANOVA with Tukey’s multiple comparisons test. *p < 0.05, **p < 0.01, ***p < 0.001. Scale bar: 100 μm.

Macrophages including tissue-resident and monocyte-derived (circulating) macrophages play distinct roles in lung tissue homeostasis and disease (Grabiec and Hussell, 2016). Tissue-resident macrophages are depleted in injury and are replaced by circulating monocytes that differentiate into macrophages with a spectrum of phenotypes (Morales-Nebreda et al., 2015). We decided to focus on macrophages derived from circulating monocytes as these drive pulmonary inflammation and fibrosis and persist in pulmonary tissue throughout the disease (Misharin et al., 2017). Therefore, we sought to assess the effect of ASTEX on bone marrow-derived macrophages (BMDMs). Exposure of BMDMs to ASTEX reduced expression of the proinflammatory cytokine IL6 and increased expression of the anti-inflammatory cytokine IL10 (Figures 4A,B). Indeed, reduction in IL6 alone is therapeutic in pulmonary fibrosis (Le et al., 2014). Another driver of pulmonary fibrosis is fibroblast activation followed by myofibroblast transdifferentiation (Kendall and Feghali-Bostwick, 2014). A primary signal for myofibroblast transdifferentiation is transforming growth factor β (TGFβ) (Chen et al., 2009). Treatment of human primary lung fibroblasts (HLF) with recombinant TGFβ induced expression of the myofibroblast marker smooth muscle actin (SMA) (Zhang et al., 1996). We decided to use SMA as a functional readout as it is necessary for focal adhesion maturation of myofibroblasts. Studies of smooth muscle actin inhibition demonstrate decreased myofibroblast development (Hinz et al., 2003; Sousa et al., 2007). Cotreatment of HLF with TGFβ and ASTEX reduces SMA expression (Figures 4C,D and Supplementary Figure 1A). Endothelial cells are another cell type that transdifferentiates into myofibroblasts upon exposure to insult; a process called endothelial to mesenchymal transition (EndMT). This pathogenic cell type is also characterized by SMA expression. To investigate the effect of ASTEX on EndMT, we exposed human umbilical vein endothelial cells (HUVECs) to IL1b and TGFβ. HUVECs exposed to these cytokines upregulated the expression of SMA (Figures 4E,F). This effect was attenuated in cells cotreated with ASTEX. Morphologically, HUVECs treated with IL1b and TGFβ had a hypertrophied tube-shaped structure typical of myofibroblasts which were attenuated in cells treated with ASTEX (Supplementary Figure 1B). Taken together, these findings demonstrate that ASTEX inhibit IL6, a major driver of inflammation in lung inflammation. Furthermore, ASTEX attenuate fibroblast activation and EndMT as seen by inhibition of smooth muscle actin. By targeting markers of inflammation and fibrosis, these findings suggest that ASTEX may exert therapeutic bioactivity in pulmonary injury.

### ASTEX Exhibit a Favorable Safety Profile in Healthy Animals

**FIGURE 5:**
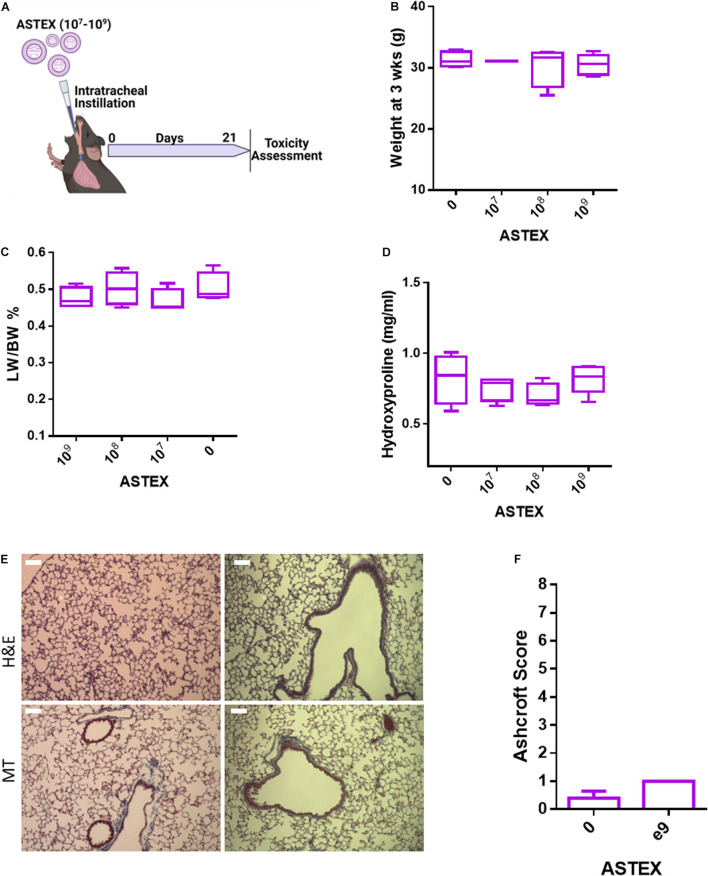
ASTEX have a favorable safety profile in healthy mice. **(A)** Design of safety study. **(B)** Animal weight at 3 weeks postintratracheal instillation of different doses of ASTEX. **(C)** Lung weight to body weight ratio of animals at 3 weeks postexposure. **(D)** Lung fibrotic content as measured by hydroxyproline context in tissue 3 weeks postexposure. **(E)** H&E staining and Masson’s trichrome of lung tissue and **(F)** tissue scoring (*n* = 4–5 animals per group) Statistical analysis was done using one way ANOVA with Tukey’s multiple comparisons test. *p < 0.05, **p < 0.01, ***p < 0.001.

Before evaluating the therapeutic efficacy of ASTEX in lung injury, we sought to evaluate the safety of intratracheal instillation of ASTEX into healthy C57BL/6 mice (Figure 5A). At 3 weeks post-ASTEX instillation, animals exposed to a range of doses showed no weight loss (Figure 5B). Furthermore, no changes were seen in lung weight to body weight ratio indicating lack of inflammatory reaction (e.g., pulmonary edema; Figure 5C). Animals treated with ASTEX developed no fibrotic burden in the lung compared with naïve animals (Figure 5D). Finally, histological analysis including hematoxylin and eosin (H&E) and Masson’s trichrome staining showed no remarkable signs of inflammation or fibrosis as evaluated by the Ashcroft score in a blinded manner (Figures 5E,F). These findings demonstrate a highly favorable safety profile of ASTEX.

### ASTEX Exert Immunomodulatory and Antifibrotic Effects in a Mouse Model of Bleomycin-Induced Lung Injury

**FIGURE 6:**
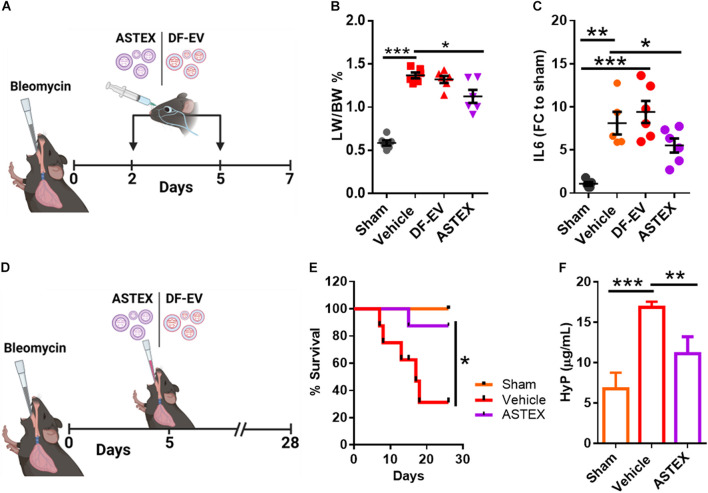
ASTEX are therapeutic in a mouse model of lung injury. **(A)** Design of the short-term bleomycin study. **(B)** Lung weight to body percent and **(C)** IL6 expression in lung tissue. **(D)** Study design for the long-term bleomycin study. **(E)** Kaplan–Meier curve showing survival of bleomycin-treated groups and sham animals (*n* = 6 animals per group) and lung fibrotic content of animals as measured by hydroxyproline content **(F)**. Statistical analysis was done using one-way ANOVA with Tukey’s multiple comparisons test. *p < 0.05, **p < 0.01, ***p < 0.001.

Having shown that ASTEX contain immunomodulatory and antifibrotic miRs, exert salutary effects in three relevant cell types *in vitro*, and exert no apparent toxic effects *in vivo*, we then investigated the therapeutic bioactivity of ASTEX in bleomycin-induced lung injury. Intratracheal (IT) instillation of bleomycin is a well-established model of lung injury. The hyperinflammatory period sustains until day 5 postexposure with an active profibrotic and injury resolution phase that sustains for 3 weeks thereafter (Kolb et al., 2020). We sought to investigate the effect of ASTEX on both short-term inflammation and long-term fibrotic development. In the first experiment, animals were given intravenous injections (retroorbital administration) of ASTEX, DF-EV, or vehicle at days 2 and 5 postbleomycin exposure and sacrificed on day 7 for analysis (Figure 6A). None of the treatments affected the weight loss seen with bleomycin exposure (Supplementary Figure 2). However, animals receiving ASTEX had attenuated lung inflammation as demonstrated by lower lung weight to body weight ratio (Figure 6B). Cell blood count (CBC) measurements from blood smears at baseline (day 2) and endpoint (day 7) showed no significant difference in overall leukocyte count (though all bleomycin-treated groups trended toward lower leukocyte count; Supplementary Figure 3A). Similarly, no differences were observed in neutrophil count (Supplementary Figure 3B). However, ASTEX-treated animals had significantly lower levels of circulating monocytes compared with vehicle-treated animals at day 7 and comparable with uninjured animals (Supplementary Figure 3C). As observed in the macrophage *in vitro* assay (Figure 4A), IL6 expression was reduced in lung tissue compared with ASTEX-treated animals compared with DF-EV or vehicle (Figure 6C). Inflammatory cytokine array analysis of lung tissue lysates identified no statistically significant differences between the groups. However, a trend toward lower proinflammatory cytokine levels including eotaxin 2, IL1a, and IL6 was observed in ASTEX-treated lung tissue compared with vehicle and DF-EV (Supplementary Figure 4). The lack of statistical significance may be due, in part, to the patchy nature of bleomycin injury in lung tissue and that in some animals one lobe is preferentially affected over the other giving rise to high variability. It may also be due to the later time point of lung collection (day 8) where inflammation is resolved, and the differences are more subtle. In a second study, animals received a single IT administration of ASTEX or vehicle at the beginning of the fibrotic phase and followed for 28 days postbleomycin exposure (Figure 6D). Animals receiving ASTEX had significantly better survival (Figure 6E) and lower fibrotic burden as shown by hydroxyproline content in lung tissue (Figure 6F). Taken together, this suggests an immunomodulatory and antifibrotic capacity of ASTEX in a bleomycin model of lung injury.

## Discussion

Pulmonary fibrosis is a progressive and deadly disease for which no curative strategy exists. EV therapy represents an advance in regenerative medicine as it is a cell-free therapeutic. ASTEX were developed based on previous investigation into the mechanism by which cells and their EVs exert therapeutic potency. Augmenting therapeutically inert fibroblasts with beta-catenin and gata4 alters the proteomic and RNA content, and the therapeutic utility of the EVs. Here, we demonstrate *in vitro* and *in vivo* evidence of the therapeutic bioactivity of ASTEX in models relevant to lung injury. As discussed earlier, the miR-92a-BMP signaling axis has been implicated in the mechanism of action of ASTEX. BMP activation has been shown to drive the salutary effects observed in cardiac, skeletal, and pulmonary injury models where ASTEX were tested. BMP activation in macrophages inhibits inflammatory activation through, in part, IL6 inhibition (Xia et al., 2011; Talati et al., 2014; Wei et al., 2018). It has been proposed that the onset of familial pulmonary arterial hypertension involves a two-hit event comprising a BMP receptor loss of function mutation and dysregulation of IL6 (Hagen et al., 2007). These findings corroborate our observation of the inhibitory effect of ASTEX on IL6 in macrophages and SMA expression in TGFβ-activated fibroblasts. Of course, miR-92a operates in a combination of other miRs and small RNAs which might function additively or synergistically.

While the present findings and mechanistic rationale suggest a therapeutic utility of ASTEX in PF, this study remains preliminary. The proposed mechanism for ASTEX function (*via* miR-92a transmission and BMP activation) has yet to be validated. Furthermore, the full anti-inflammatory effect of ASTEX in the inflammatory response remains to be fully elucidated. Another limitation of this study is the limited scope of the investigation. For instance, we have not investigated the effect of ASTEX on resident (bronchioalveolar) macrophages. These findings will also need to be validated *in vivo* to ensure the generalizability of *in vitro* findings. Finally, while we have investigated the anti-inflammatory and antifibrotic properties of ASTEX, the effect of this therapeutic on the alveolar progenitor niche has not been investigated. Therefore, future studies will explore the current findings in-depth and broaden the scope of the effect of ASTEX on pulmonary tissue and, more specifically, therapeutically relevant pathways. Increasing understanding of how EVs like ASTEX function to repair pulmonary tissue offers a new avenue of therapeutic development for this and other diseases for which no curative strategy exists.

## Materials and Methods

### Animal Subjects

All procedures related to animals have been conducted under approved Institutional Animal Care and Use Committee protocols.

#### Activated, Specialized Tissue Effector Cells Preparation

Engineered fibroblasts (ASTECs) and their EVs (ASTEX) were prepared as described in detail previously (Ibrahim et al., 2019). Briefly, primary neonatal skin fibroblasts cultured in 10% FBS, 50 μg/ml gentamicin, and 2 mmol/l L-glutamine in Iscove’s modified Dulbecco’s medium (complete medium). Cells were transduced (MOI: 20) with lenti-viruses containing transgenes for ctnnb1 (Santa Cruz Biotechnology, Dallas, TX, United States) and gata4 (AMSBIO, Abingdon, United Kingdom). Following 1 week in selection (puromycin and blasticidin for ctnnb1 and gata4, respectively), cells are expanded and banked for future use.

#### Activated Specialized Tissue Effector Extracellular Vesicles Preparation

To produce EVs from ASTECs (ASTEX), cells are expanded in a tri-layer flask (Thermo Fisher Scientific, Waltham, MA, United States) in complete media. When cells reach confluence, complete media are removed, and cells are washed with Iscove’s modified Dulbecco’s medium (IMDM) to remove FBS contaminants. Cells are then conditioned in IMDM for 15 days. Prior to ASTEX isolation, conditioned media are cleared from cellular debris through a 0.45-μm vacuum filter. ASTEX are isolated using ultrafiltration using a 100-kDa centrifugal filter.

### Bleomycin Model

Fourteen-week-old male C57BL/6 mice were instilled with 0.7 U/kg (retro-orbitally for short-term study) or 1.5 U/kg (intratracheally) for long-term study of bleomycin (MilliporeSigma, Burlington, MA, United States) exposure. Animals were sacrificed at day 7 or 28 for the long- and short-term studies, respectively.

### Lung Fibroblast Model

Primary adult human lung fibroblasts [American Type Culture Collection (ATCC)] were maintained in fibroblast basal medium (ATCC) and supplemented with fibroblast growth kit-low serum (ATCC). Cells were expanded for three passages before performing TGFβ exposure assay. Briefly, cells were exposed to 5 ng/ml of recombinant TGFβ (Thermo Fisher Scientific). Cells were also cotreated with ASTEX at a cell to ASTEX ratio of 1:100. Cells were incubated for 48 h prior to Western blot or flow cytometry analysis.

### Human Umbilical Vein Endothelial Cell Endothelial to Mesenchymal Transition Assay

Human umbilical vein endothelial cells (HUVECs: ATCC) were maintained in endothelial cell medium supplemented with endothelial growth kit (ATCC). Cells were expanded for three passages before exposure to 5 ng/ml of recombinant TGFβ (Thermo Fisher Scientific) and 1 ng/ml of IL1b (Thermo Fisher Scientific) for 5 days. Cells were treated at day 0 with vehicle or ASTEX at a cell to ASTEX ratio of 1:100.

#### Macrophage Isolation Assay and Model

Macrophages were prepared by isolating bone marrow cells from 3-month-old female Wistar Kyoto rats. Monocytes were purified and differentiated into BMDM by culturing with recombinant M-CSF (20 ng/ml; Life Technologies, Carlsbad, CA, United States). Whole bone marrow cells were collected *via* aspiration with ice-cold phosphate-buffered saline (PBS) and filtered using a 70-μm cell strainer and centrifuged at 400 × *g* for 10 min. Red blood cells were lysed out using 10 ml ACK buffer (Gibco, Waltham, MA, United States) for 30 s followed by quenching with complete media (IMDM + 10% FBS + 20 ng/ml M-CSF). Cells were centrifuged and resuspended in complete media and cultured for 5 days at 37°C with 20% O_2_ and 5% CO_2_. Cells were then seeded into six-well plates at 6–8.0*e*10 (Diamantopoulos et al., 2018) cells/well. Cells were incubated. Fresh complete media were exchanged on day 5, and cells were monitored for confluence. EVs were administered once BMDM cultures reached ∼75% confluence. Serum concentration was reduced to 1% during assays to allow EV uptake. BMDMs were cultured with EVs over night before RNA was isolated for analysis.

### EV Proteomics

#### Chemicals and Instrumentation

DL-Dithiothreitol (DTT), iodoacetamide (IAA), formic acid (FA), and acetonitrile (ACN) were purchased from Sigma-Aldrich (St. Louis, MO, United States). Trypsin from bovine pancreas was purchased from Promega (Madison, WI, United States). Ultrapure water was prepared from a Millipore purification system (Billerica, MA, United States). An Ultimate 3000 nano-UHPLC system coupled with a Q Exactive^TM^ HF mass spectrometer (Thermo Fisher Scientific) with an ESI nanospray source.

#### Sample Preparation

(1) Take 100 μg protein per sample in the ultrafiltration tube. Transfer the solution into Microcon devices YM-10 (Millipore). The device was centrifuged at 12,000 rpm at 4°C for 10 min. Subsequently, 200 μl of 50 mM ammonium bicarbonate were added to the concentrate followed by centrifugation and repeat once.
(2) After reduced by 10 mM DTT at 56°C for 1 h and alkylated by 20 mM IAA at room temperature in the dark for 1 h, the device was centrifuged at 12,000 rpm at 4°C for 10 min and wash once with 50 mM ammonium bicarbonate.
(3) Add 100 μl of 50 mM ammonium bicarbonate and free trypsin into the protein solution at a ratio of 1:50, and the solution was incubated at 37°C overnight.
(4) Finally, the device was centrifuged at 12,000 rpm at 4°C for 10 min; 100 μl of 50 mM ammonium bicarbonate was added into the device and centrifuged, and then repeat once.
(5) Lyophilize the extracted peptides to near dryness. Resuspend peptides in 20 μl of 0.1% formic acid before liquid chromatography coupled to tandem mass spectrometry (LC-MS/MS) analysis.

#### Nano-LC-MS/MS Analysis

##### Nano-LC

Nanoflow UPLC: Ultimate 3000 nano-UHPLC system (Thermo Fisher Scientific, United States).

Nanocolumn: trapping column (PepMap C18, 100Å, 100 μm × 2 cm, 5 μm) and an analytical column (PepMap C18, 100 Å, 75 μm × 50 cm, 2 μm).

Loaded sample volume: 1 μg.

Mobile phase: A: 0.1% formic acid in water; B: 0.1% formic acid in 80% acetonitrile.

Total flow rate: 250 nl/min.

LC linear gradient: from 2 to 8% buffer B in 3 min, from 8 to 20% buffer B in 56 min, from 20 to 40% buffer B in 37 min, then from 40 to 90% buffer B in 4 min.

##### Mass Spectrometry

The full scan was performed between 300 and 1,650 *m*/*z* at the resolution 60,000 at 200 *m*/*z*, the automatic gain control target for the full scan was set to 3e6. The MS/MS scan was operated in Top 20 mode using the following settings: resolution 15,000 at 200 *m*/*z*; automatic gain control target 1e5; maximum injection time 19 ms; normalized collision energy at 28%; isolation window of 1.4 Th; charge sate exclusion: unassigned, 1, >6; dynamic exclusion 30 s.

#### Data Analysis

Six raw MS files were analyzed and searched against human protein database based on the species of the samples using Maxquant (1.6.2.14). The parameters were set as follows: the protein modifications were carbamidomethylation (C) (fixed), oxidation (M) (variable); the enzyme specificity was set to trypsin; the maximum missed cleavages were set to 2; the precursor ion mass tolerance was set to 10 ppm; and MS/MS tolerance was 0.6 Da.

EVs were lysed using RIPA buffer followed by acetone precipitation and reduction using 1 mM tris(2-carboxyethyl) phosphine (TCEP). Samples were then alkylated with 5 mM iodoacetamide and digested with ∼1:40 trypsin-LyC (Promega, Madison, WI, United States), desalted, and speed vac to total dryness. Liquid chromatography MS/MS was done using a Dionex Ultimate 3000 Nano-LC coupled with an Orbitrap Q Exactive^TM^ mass spectrometer (Thermo Fisher Scientific, USA) with an ESI nanospray source. Mobile phase A comprised 0.1% aqueous formic acid and mobile phase B of 0.1% formic acid in acetonitrile. Peptides were then loaded onto the analytical column [100 μm × 10 cm in-house-made column packed with a reversed-phase ReproSil-Pur C18-AQ resin (3 μm, 120 Å, Dr. Maisch GmbH, Germany)] at a flow rate of 600 nl/min using a linear AB gradient composed of 6–30% B for 38 min, 30–42% B for 10 min, 42–90% B for 6 min, and 90% B elution for 6 min. The temperature was set to 40°C for both columns. Nanosource capillary temperature was set to 270°C and spray voltage set to 2.2 kV. The Orbitrap Q Exactive^TM^ operated in a data-dependent mode, where the five most intensive precursors were selected for subsequent fragmentation. Resolution for the precursor scan (*m*/*z* 300–1,800) was set to 70,000 at *m*/*z* 400 with a target value of 1 × 106 ions. The MS/MS scans were also acquired in the orbitrap with a normalized collision energy setting of 40 for HCD.

Raw MS files were then analyzed and cross-referenced against the human protein database based on the species of the samples using Maxquant (1.5.6.5). The parameters were set as follows: the protein modifications were carbamidomethylation (C) (fixed) and oxidation (M) (variable); the enzyme specificity was set to trypsin; the maximum missed cleavages were set to 2; the precursor ion mass tolerance was set to 10 ppm; and MS/MS tolerance was 0.6 Da. Only high confident identified peptides were chosen for downstream protein identification analysis. The listed common secreted proteins were classified using Panther (Protein Analysis Through Evolutionary Relationships^1^). To glean relevant biological processes, transcription factors, and FunRich (Functional Enrichment Analysis Tool) to explore protein classification, molecular function, and pathways.

### RNA Isolation and qRT-PCR

Total RNA was isolated using miRNeasy Mini Kit (Qiagen, Hilden, Germany) for cells or Urine Exosome RNA Isolation Kit (Norgen Biotek Corp., Thorold, ON, Canada) for exosomes. Reverse transcription was performed using High-Capacity RNA to cDNA (Thermo Fisher Scientific) or Taqman^®^ microRNA Reverse Transcription Kit (Applied Biosystems, Waltham, MA, United States). Real-time PCR was performed using Taqman Fast Advanced Master Mix and the appropriate TaqMan^®^ Gene Expression Assay (Thermo Fisher Scientific). Samples were measured using Applied Biosystems 7800HT fast Real-Time PCR system. Each reaction was performed in triplicate samples and adjusted using hprt1 and u6 for mRNA and miRs, respectively. The gene expression assays/microRNAs used in this study were as follows (Thermo Fisher Scientific):

**Table:** 

**Assay names**	**Species**	**Assay numbers**
hsa-miR182-5p	Human	002334
hsa-miR183-5p	Human	002269
hsa-miR-92a-3p	Human	000431
IL6	Rat	Rn01410330_m1
IL10	Rat	Rn01483988_g1
IL6	Mouse	Mm00446190

### Flow Cytometry

Cells were harvested and counted (2 × 10^5^ cells per condition) and washed with 1% bovine serum albumin (BSA) in 1 × PBS and stained with the appropriate antibody (BD Pharmingen, San Diego, CA, United States) for 1 h at 4°C. The cells were then washed again and resuspended in 1% BSA in 1× PBS. BD Cytofix/Cytoperm^TM^ kit was used for cell permeabilization before staining. Flow cytometry was performed using the Sony SA3800 Spectral Cell Analyzer.

#### Exosome Preparation and Collection

Exosomes were harvested from transduced fibroblasts at passage 16 under hypoxia conditioning. Briefly, cells were grown in 636 cm^2^ CellStack chambers (Corning, Corning, NY, United States) until confluence. The flask was then washed three times with phenol-free IMDM and conditioned in the same for 15 days under 20% O_2_. Conditioned media were collected, pooled, and filtered through 0.45 μm filter to remove apoptotic bodies and cell debris and frozen at −80°C for later use. Extracellular vesicles were isolated using centrifuge-based ultrafiltration with a molecular weight cutoff of 100 kDa (MilliporeSigma). Isolated EVs were quantified using Malvern Nanosight NS300 Instrument (Malvern Instruments, Malvern, UK) with the following acquisition parameters: camera levels of 15, detection level less than or equal to 5, number of videos taken 4, and video length of 30 s.

### Hydroxyproline Assay

Hydroxyproline content in lung tissue was quantified chemically using a Hydroxyproline Assay Kit (Sigma-Aldrich) per manufacturer’s instructions.

### Mouse Inflammatory Cytokine Array

Expression of inflammatory markers in lung tissue lysates from animals exposed to bleomycin (including those also treated with ASTEX, DF-EVs, or vehicle) or sham control (day 8 postexposure) were measured using a Quantibody^®^ Mouse Inflammation Array 1 Kit (RayBiotech, Peachtree Corners, GA, USA) per manufacturer’s instructions.

### Western Blot

Membrane transfer was performed using the Turbo Transfer System (Bio-Rad, Hercules, CA, United States) after gel electrophoresis. The following antibody staining was then applied and detected by SuperSignal^TM^ West Pico PLUS Chemiluminescent Substrate (Thermo Fisher Scientific). To prove for SMA, antimouse human/mouse/rat antibody (MAB1420-SP; R&D Systems, Minneapolis, MN, United States) was used.

### Exosome RNA Sequencing

#### Library Preparation and Sequencing: miRNAs

The miRNA sequencing library was prepared using the QIASeqTM miRNA Library Kit (Qiagen). Total RNA was used as the starting material. A preadenylated DNA adapter was ligated to the 3′ ends of miRNAs, followed by ligation of an RNA adapter to the 5′ end. A reverse-transcription primer containing an integrated Unique Molecular Index (UMI) was used to convert the 3′/5′-ligated miRNAs into cDNA. After cDNA cleanup, indexed sequencing libraries were generated *via* sample indexing during library amplification, followed by library cleanup. Libraries were sequenced on a NextSeq 500 (Illumina, San Diego, CA, United States) with a 1 × 75 bp read length and an average sequencing depth of ∼10 M reads/sample.

#### Data Analysis

The demultiplexed raw reads were uploaded to GeneGlobe Data Analysis Center (Qiagen) at https://www.qiagen.com/us/resources/geneglobe/, for quality control, alignment, and expression quantification. Briefly, 3′ adapter and low-quality bases were trimmed off from reads first using cutadapt (version 1.13) with default settings, then reads with less than 16 bp insert sequences or with less than 10 bp UMI sequences were discarded (Martin, 2011). The remaining reads were collapsed to UMI counts and aligned to miRBase (release v21) mature and hairpin databases sequentially using Bowtie v1.2 (Langmead et al., 2009). The UMI counts of each miRNA molecule were counted, and the expression of miRNAs was normalized based on total UMI counts for each sample.

#### Total RNA Sequencing

Library construction was performed using the SMARTer^®^ Stranded Total RNA-Seq Kit (Takara Bio USA, Inc., Mountain View, CA, United States). Briefly, total RNA samples were assessed for concentration using a Qubit fluorometer (Thermo Fisher Scientific) and for quality using the 2100 Bioanalyzer (Agilent Biotechnologies, Santa Clara, CA, United States). Total RNA was converted to cDNA, and adapters for Illumina sequencing (with specific barcodes) were added through PCR using only a limited number of cycles. The PCR products were purified, and then ribosomal cDNA was depleted. The cDNA fragments were further amplified with primers universal to all libraries. Lastly, the PCR products were purified once more to yield the final library. The concentration of the amplified library was measured with a Qubit fluorometer, and an aliquot of the library was resolved on the Bioanalyzer. Sample libraries were multiplexed and sequenced on a NextSeq 500 platform (Illumina) using 75 bp single-end sequencing. On average, 50 million reads were generated from each sample.

#### Data Analysis

Raw reads obtained from RNA-Seq were aligned to the transcriptome using STAR (version 2.5.0) (Dobin et al., 2013)/RSEM (version 1.2.25) (Li and Dewey, 2011) with default parameters, using human GRCh38 (or mouse CRCm38) transcriptome reference downloaded from http://www.gencodegenes.org. Expression counts for each gene [transcripts per million (TPM)] in all samples were normalized by the sequencing depth.

### Statistical Analysis

Statistical comparisons between two groups were made using an independent two-tailed independent Student’s *t*-test with a 95% CI. Comparisons between three or more groups were done using one-way ANOVA with Tukey’s or Dunnett’s test for multiple comparisons. Calculations were done using Graphpad Prism 6.0 software.

## Data Availability Statement

The data presented in this study are deposited in Exocarta under accession ID ExoCarta_312.

## Ethics Statement

All procedures animal-related work was conducted under approved Institutional Animal Care and Use Committee (IACUC) protocols at Cedars Sinai Medical Center.

## Author Contributions

AI and AC conceived the idea, performed the experiments, and wrote the manuscript. AA, CL, KP, AMo, and KJ-U performed the experiments and analyzed the data. AMa analyzed the data. EM conceived the idea and analyzed the data. AGI conceived the idea, performed the experiments, analyzed the data, and wrote the manuscript. All authors contributed to the article and approved the submitted version.

## Conflict of Interest

EM owns founder stock in Capricor Therapeutics. The remaining authors declare that the research was conducted in the absence of any commercial or financial relationships that could be construed as a potential conflict of interest.

## Publisher’s Note

All claims expressed in this article are solely those of the authors and do not necessarily represent those of their affiliated organizations, or those of the publisher, the editors and the reviewers. Any product that may be evaluated in this article, or claim that may be made by its manufacturer, is not guaranteed or endorsed by the publisher.

## Footnotes

1
http://pantherdb.org

## Supplementary Material

Supplementary Figure 1 **(A)** Flow cytometry of human lung fibroblasts treated with TGFβ only (vehicle) or TGFβ with ASTEX. **(B)** Phase contract images of HUVECs, treated with TGFβ + IL1b only (vehicle) or TGFβ + IL1b with ASTEX six days postexposure (scale bar: 100 μm).

Supplementary Figure 2 Change in animal weight in animals treated with bleomycin (vehicle, DF-EV, or ASTEX) and sham (*n* = 5 animals/group). Statistical analysis was done using one-way ANOVA with Dunnett’s multiple comparisons test. ^∗^*p* < 0.05, ^∗∗^*p* < 0.01, ^∗∗∗^*p* < 0.001.

Supplementary Figure 3 Cell blood count of animals exposed to bleomycin (vehicle, DF-EVs, and ASTEX) or sham. **(A,B)** Total white blood cell count, **(C,D)** neutrophils, and **(E,F)** monocytes (*n* = 5 animals per group). Statistical analysis was done using one-way ANOVA with Dunnett’s multiple comparisons test. ^∗^*p* < 0.05, ^∗∗^*p* < 0.01, ^∗∗∗^*p* < 0.001.

Supplementary Figure 4 Inflammatory cytokine array of mouse lung tissue lysates. Lysates were collected from lung tissue of bleomycin and sham-treated groups 8 days postbleomycin exposure (*n* = 4 mice per group). Statistical analysis was done using one-way ANOVA with Tukey’s multiple comparisons test.
