# Supplementary figures and images for: Engineered Fibroblast Extracellular Vesicles Attenuate Pulmonary Inflammation and Fibrosis in Bleomycin-Induced Lung Injury
Source: Front Cell Dev Biol. 2021 Sep 23;9:733158. doi: 10.3389/fcell.2021.733158 (PMC8512699; doi:10.3389/fcell.2021.733158)

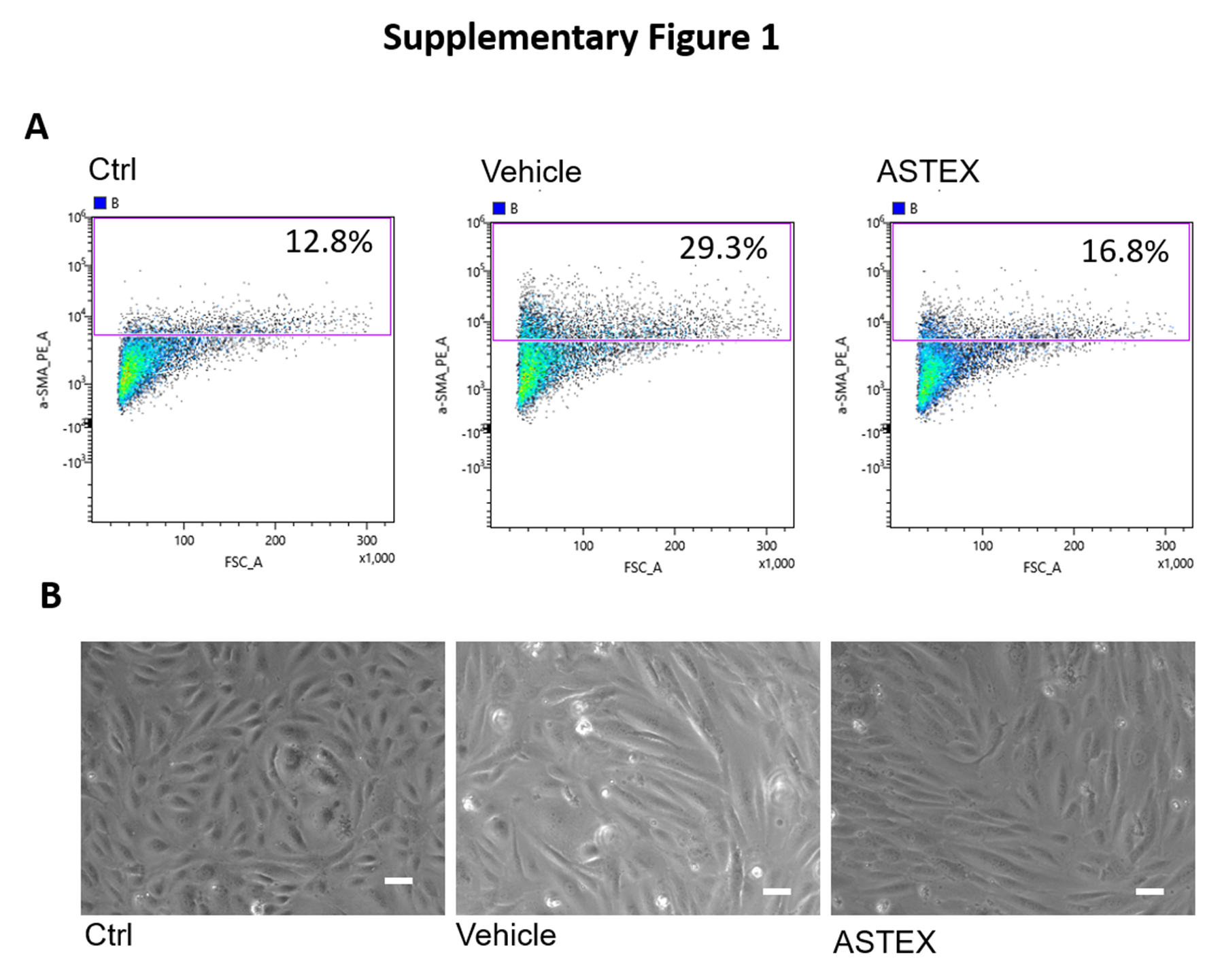

Supplement: Supplementary Figure 1 — (A) Flow cytometry of human lung fibroblasts treated with TGFβ only (vehicle) or TGFβ with ASTEX. (B) Phase contract images of HUVECs, treated with TGFβ + IL1b only (vehicle) or TGFβ + IL1b with ASTEX six days postexposure (scale bar: 100 μm). [file Image_1.tif]

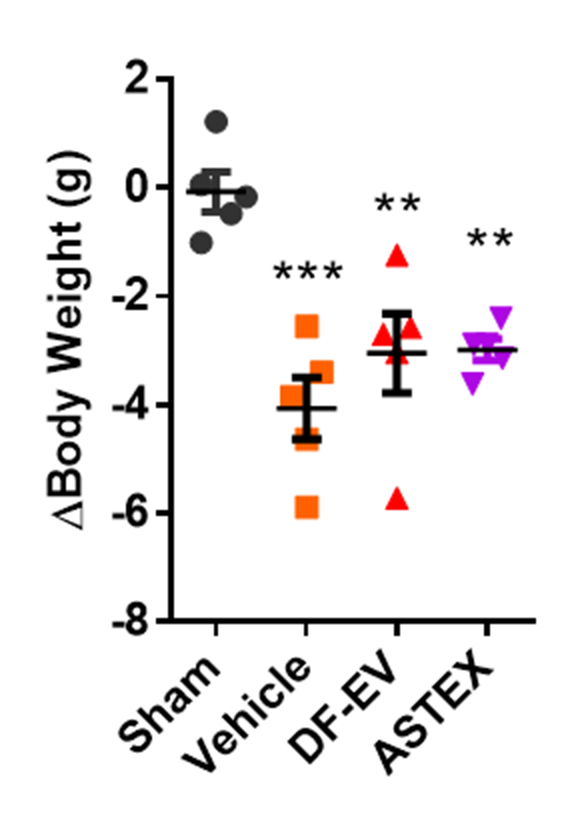

Supplement: Supplementary Figure 2 — Change in animal weight in animals treated with bleomycin (vehicle, DF-EV, or ASTEX) and sham (n = 5 animals/group). Statistical analysis was done using one-way ANOVA with Dunnett’s multiple comparisons test. ∗p < 0.05, ∗∗p < 0.01, ∗∗∗p < 0.001. [file Image_2.tif]

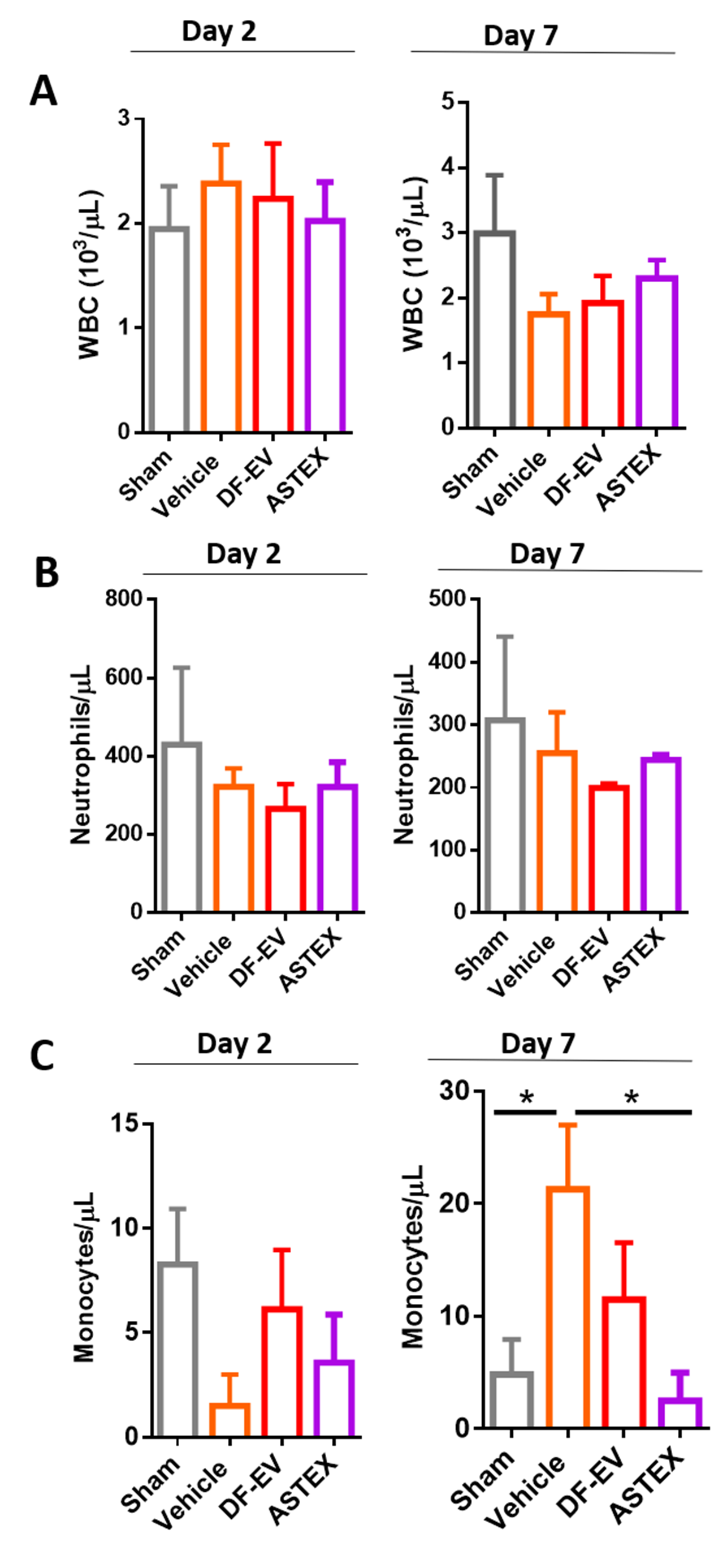

Supplement: Supplementary Figure 3 — Cell blood count of animals exposed to bleomycin (vehicle, DF-EVs, and ASTEX) or sham. (A,B) Total white blood cell count, (C,D) neutrophils, and (E,F) monocytes (n = 5 animals per group). Statistical analysis was done using one-way ANOVA with Dunnett’s multiple comparisons test. ∗p < 0.05, ∗∗p < 0.01, ∗∗∗p < 0.001. [file Image_3.tif]

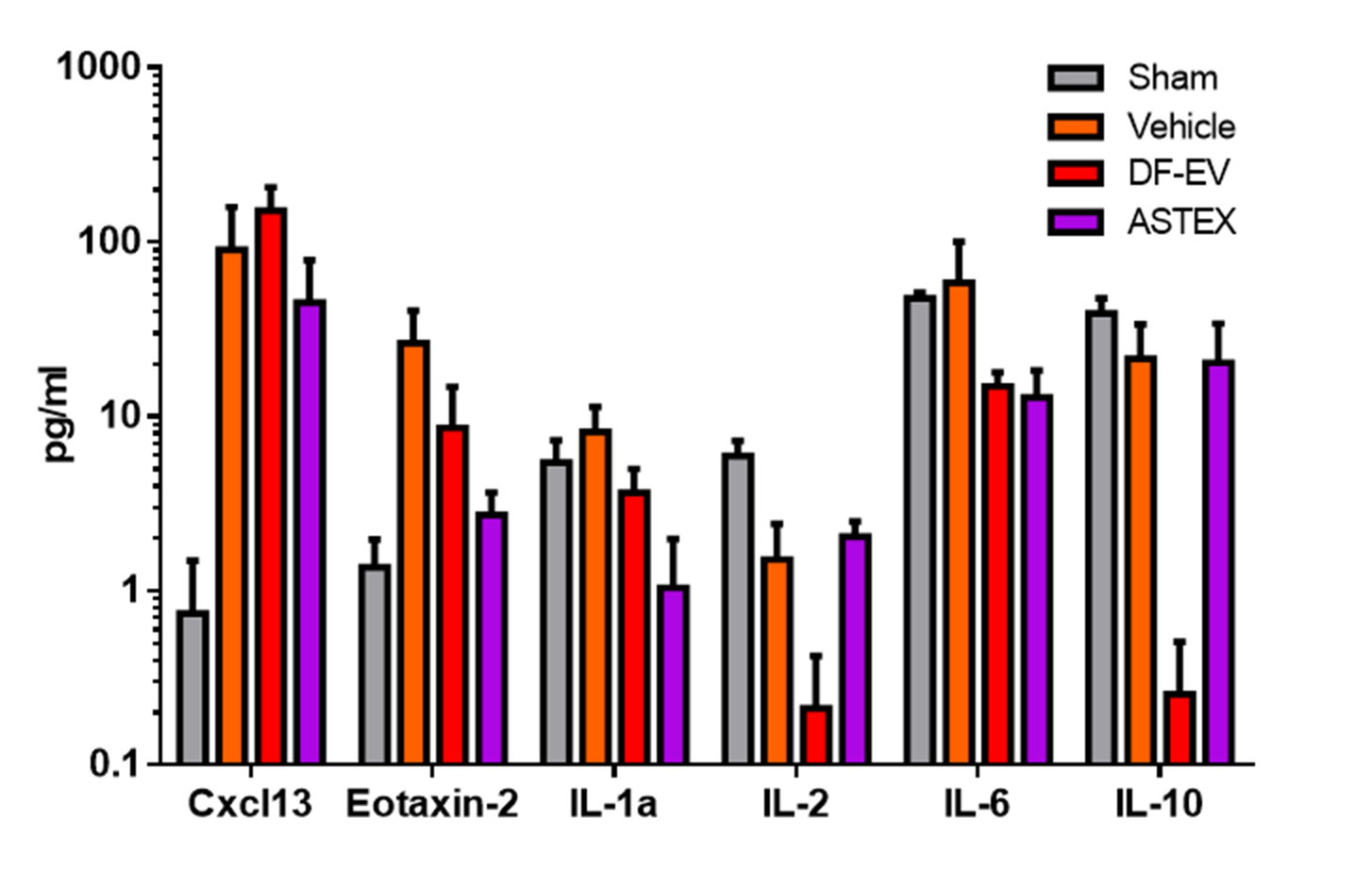

Supplement: Supplementary Figure 4 — Inflammatory cytokine array of mouse lung tissue lysates. Lysates were collected from lung tissue of bleomycin and sham-treated groups 8 days postbleomycin exposure (n = 4 mice per group). Statistical analysis was done using one-way ANOVA with Tukey’s multiple comparisons test. [file Image_4.tif]
